# Inflammatory cytokines induce vascular endothelial growth factor-C expression in melanoma-associated macrophages and stimulate melanoma lymph node metastasis

**DOI:** 10.3892/ol.2014.2297

**Published:** 2014-06-30

**Authors:** SILVIA PEPPICELLI, FRANCESCA BIANCHINI, LIDO CALORINI

**Affiliations:** Department of Experimental and Clinical Biomedical Sciences, University of Florence, Florence 50134, Italy

**Keywords:** inflammatory cytokines, VEGF-C, macrophages, melanoma cells

## Abstract

Lymph node colonization by tumor cells is one of the key determinants of melanoma staging and prognosis, and tumor-associated macrophages (TAMs) are the predominant type of inflammatory cell in the tumor environment which secretes vascular endothelial growth factor (VEGF)-C, the most potent lymphangiogenic growth factor. In the present study, to elucidate the mechanism involved in VEGF-C expression in TAMs, murine peritoneal macrophages were co-cultivated with syngeneic B16 melanoma cells to mimic the reciprocal interactions between tumor cells and macrophages found in spontaneous tumors. In the present study, upon contact with tumor cells, macrophages were found to express a higher level of VEGF-C which was associated with an increase in the expression of IL-1β and TNF-α and their receptors. Antibodies against the IL-1β and TNF-α receptors were added to media that had been conditioned by the macrophage-tumor cell co-cultures and inhibition of VEGF-C was observed in macrophages co-cultivated with the tumor cells. Furthermore, when IL-1β and TNF-α were used at a non-toxic level, they enhanced peritoneal lymph node colonization by melanoma cells. Thus, in the present study, macrophagic IL-1β and TNF-α were observed to promote VEGF-C expression in TAMs, as well as melanoma lymph node metastasis, suggesting that inhibiting the signaling between tumor cells and TAMs may be required to inhibit lymphangiogenesis and lymph node metastasis.

## Introduction

The extent of lymph node involvement is one of the major determinants for the staging and prognosis of melanoma (1). Until recently, lymph node colonization by tumor cells was proposed to be a passive process involving tumor cell spread through pre-existing afferent lymphatic vessels. However, it is now well established that tumor cells, as inflammatory cells of the tumor environment, contribute to lymphatic dissemination through the *de novo* formation of lymphatic capillaries, a phenomenon termed lymphangiogenesis (2–4). Among the members of the vascular endothelial growth factor (VEGF) family, VEGF-C has been identified as the major lymphangiogenic growth factor required for lymphatic development (5,6). VEGF-C-overexpressing tumors not only exhibit an increase in intratumoral lymphangiogenesis, but also an increased number of functional peritumoral lymphatic vessels (7). Furthermore, patients with tumors expressing high levels of VEGF-C have been reported to be more likely to have advanced disease and lymph node metastasis compared with those with tumors expressing low levels of VEGF-C (8). Among the inflammatory host cells, tumor-associated macrophages (TAMs) have been found to release high levels of VEGF-C (9) and VEGF-C has been reported to promote macrophage recruitment, in addition to lymphangiogenesis, in VEGF-C-overexpressing human melanoma cells transplanted into nude mice (10). Kerjaschki (11) demonstrated that macrophages promote lymphangiogenesis either through transdifferentiating and incorporating into the endothelial layer or through stimulating the proliferation of pre-existing local lymphatic endothelial cells (11).

The present study aimed to elucidate the mechanisms involved in VEGF-C expression in TAMs, in order to identify novel approaches to inhibit lymph node dissemination. *In vivo* tumor cell-macrophage interactions were mimicked through co-culturing B16 murine melanoma cells with syngeneic peritoneal macrophages.

## Materials and methods

### Materials

Unless specified, all reagents were obtained from Sigma-Aldrich (St. Louis, MO, USA). All products for reverse transcription polymerase chain reaction (RT-PCR) analysis were purchased from Promega Corporation (Madison, WI, USA). Recombinant human and murine cytokines were purchased from PeproTech (Rocky Hill, NJ, USA).

### Cell lines and culture conditions

In the present study, a clone isolated from a B16-F10 melanoma cell line (F10-M3 cells), kindly supplied by Dr. S. Gattoni-Celli (Medical University of South Carolina, Charleston, SC, USA), was used. Cells were cultivated in Dulbecco’s modified Eagle’s medium (DMEM 4500; Gibco-BRL, Grand Island, NY, USA) supplemented with 10% fetal calf serum (Boehringer Ingelheim, Ingelheim, Germany), at 37°C in a humidified atmosphere containing 10% CO_2_. Cells were harvested from subconfluent cultures through incubation with a trypsin-EDTA solution and propagated every three days. B16 melanoma cells were exposed to TNF-α (400, 800 or 1200 U/ml) and/or IL-1β (300, 600 or 900 U/ml) for 24 h and H_2_O_2_ (200 uM) for 4 h. Cell viability was determined using the trypan blue exclusion test. Cultures were periodically monitored for mycoplasma contamination using Chen’s fluorochrome test (12).

### Macrophage cultures and macrophage-tumor cell co-cultures

Cultures of inflammatory macrophages were established from peritoneal exudates collected through lavage from C57Bl/6 mice (Charles River Laboratories, Wilmington, MA, USA) between six and eight weeks old, that had been injected intraperitoneally with 1 ml 3% thioglycollate broth 3–4 days previously (13). Co-cultures were prepared through seeding tumor cells on macrophage monolayers at a 1:1 ratio at a density of 250×10^3^ cells/cm^2^. This cell density was selected based on previous experiments and in order to establish close contact between the macrophages and the tumor cells (14).

Macrophage monolayers incubated for 2 h in DMEM 4500 containing 250 μg/ml bovine serum albumin were exposed to the following cytokines: Interferon γ (50 U/ml), TNF-α (50 ng/ml), transforming growth factor (TGF)-β (25 ng/ml), IL-4 (100 U/ml), IL-10 (10 ng/ml), lipopolysaccharide (10 ng/ml) or IL-1β (100 U/ml). For antibody treatment, tumor cell-macrophage co-cultures were exposed to either 1 μg/ml rabbit anti-mouse polyclonal anti-IL-1β receptor 1 (sc-689; Santa Cruz Biotechnology, Inc., Santa Cruz, CA, USA) and hamster anti-mouse monoclonal anti-TNF R1-p55 (sc-12746; Santa Cruz Biotechnology, Inc.) antibodies or irrelevant IgG (Santa Cruz Biotechnology, Inc.) for 24 h.

### RNA isolation and RT-PCR

Total RNA was extracted from cells using TRI Reagent^®^. The quantity and purity of the RNA was determined spectrophotometrically. Complementary DNA (cDNA) synthesis was performed through incubating 1 μg total RNA with 4 U/μl M-MLV reverse transcriptase. Aliquots of 5 μl cDNA were used for the PCR amplification. The RT-PCR reactions were performed in a 50 μl reaction volume containing specific primers (Table I) and 0.1 U/μl GoTaq^®^ Polymerase using a Perkin-Elmer Thermal cycler. Aliquots of 10 μl PCR reaction were applied to a 2% agarose gel, electrophoresed and visualized. cDNA products were analyzed on the basis of standard PCR markers.

### In vivo experiments

Subconfluent cultures of B16 melanoma cells were harvested using EDTA/trypsin solution, centrifuged at 200 × g and resuspended in DMEM medium at 2×10^6^ cells/ml. A total of 0.5 ml cell suspension was injected into the peritoneal cavity of syngeneic C57Bl/6 mice. Mice were sacrificed by cervical dislocation after seven days and the peritoneal lymph nodes were removed. Lymph node colonization by tumor cells was determined using a dissecting microscope, with lymph nodes which were colonized by murine melanoma cells observed to be enlarged and brown for melanin. B16 melanoma cell-colonized lymph nodes were weighed and formalin fixed (5% in phosphate-buffered saline) to assess the tumor burden, as well as for histological examination following hematoxylin and eosin staining. *In vivo* experiments were performed in agreement with the national guidelines and approved by the ethical committee of Animal Welfare Office of Italian Work Ministry (Rome, Italy) and conform to the legal mandates and Italian guidelines for the care and maintenance of laboratory animals

### Statistical analysis

Statistical analysis was conducted using the SPSS software, version 16.0 (SPSS, Inc, Chicago, IL, USA). Densitometric data are expressed as the mean ± standard error of the mean depicted by vertical error bars from at least three independent experiments. Statistical analyses of the data were performed using Student’s t-test and P≤0.05 was considered to indicate a statistically significant difference.

## Results

### Inflammatory cytokine and VEGF-C expression in macrophage cultures and macrophage-tumor cell co-cultures

**Figure 1:**
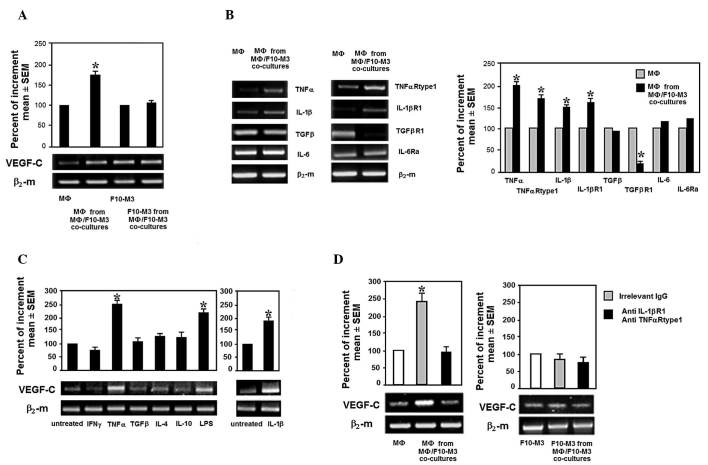
Inflammatory cytokines and VEGF-C expression in murine peritoneal macrophages co-cultivated with syngeneic B16 melanoma cells. (A) VEGF-C mRNA expressed by C57Bl/6 thioglycollate-elicited macrophages and B16 cells collected from standard cultures or co-cultures. (B) mRNA expression of TNF-α, IL-1β, IL-6, TGF-β and their receptors in murine macrophages grown with tumor cells, as well as the relative densitometric analyses. (C) VEGF-C mRNA expressed by murine thioglycollate-elicited macrophages stimulated *in vitro* by several pro- and anti-inflammatory cytokines and growth factors. (D) VEGF-C mRNA expression in macrophages grown in media conditioned by macrophage-tumor cell co-cultures in the absence or presence of anti-IL-1βR1 and -TNF-αR1 antibodies. Densitometric analyses were performed using Image J software and standardized to β2-microglobulin mRNA expression. ^*^P<0.05 vs. control. VEGF, vascular endothelial growth factor; IL, interleukin; TNF, tumor necrosis factor; TGF, transforming growth factor; β2-m, β2-microglobulin; R, receptor; SEM, standard error of the mean.

In order to mimic the multiple interactions that tumor cells establish with macrophages during tumor growth, F10-M3 murine melanoma cells were co-cultured with syngeneic C57Bl/6 thioglycollate-elicited peritoneal macrophages at a high cell density. After 24 h, the tumor cells were removed from the co-cultures and collected. Macrophages adherent to the tissue culture dishes were analyzed for VEGF-C expression. Trypan blue exclusion test revealed that the tumor cells and macrophages had 95–97% cell viability. The number of tumor cells present in the macrophage preparations was <0.1%, demonstrating that tumor cells are easily removed from culture dishes using trypsin-EDTA solution, whereas adherent macrophages require specific treatment, for example using the anesthetic lidocaine, or scraping (15). Fig. 1A shows that VEGF-C expression in macrophages is stimulated through contact with tumor cells, whereas there is no significant change in VEGF-C expression in the tumor cells following co-cultivation with macrophages.

Based on the finding that macrophages express tumor promoting properties associated with changes in cytokine production upon contact with tumor cells and that VEGF-C is stimulated by cytokines, the levels of a series of inflammatory cytokines and their receptors were assessed in macrophages co-cultivated with tumor cells. Macrophages exposed to tumor cells were observed to express an enhanced level of IL-1β, TNF-α and theirs receptors, and a reduced level of TGF-β receptor 1 (Fig. 1B). As shown in Fig. 1C, exogenous TNF-α and IL-1β were found to promote VEGF-C expression in murine macrophages. Furthermore, antibodies against IL-1β and TNF-α receptors added to media conditioned by macrophage/tumor cell co-cultures were observed to inhibit the enhanced expression of VEGF-C in macrophages (Fig. 1D).

### Effect of TNF-α and IL-1β on murine melanoma cell viability and lymph node metastasis

**Figure 2:**
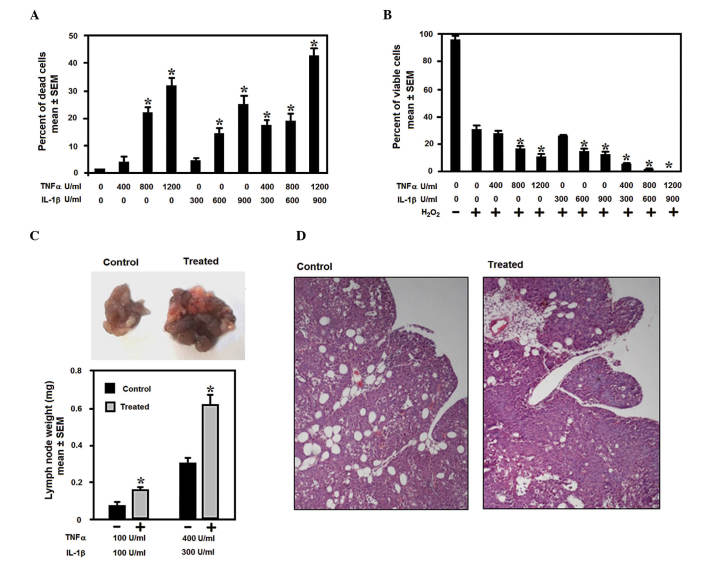
Effect of TNF-α and IL-1β on murine melanoma cell viability and lymph node metastasis. (A) B16 cells were exposed to increasing concentrations of TNF-α and IL-1β and harvested for trypan blue exclusion assay. Data are presented as the percentage of dead cells in the total cell number. (B) Cells pretreated with increasing concentrations of TNF-α and IL-1β were exposed to H_2_O_2_, a pro-apoptotic signal. Data are presented as the percentage of viable cells in the total cell number. (C) Enhanced lymph node colonization of B16 cells stimulated by TNF-α and IL-1β. B16 cells were stimulated *in vitro* by TNF-α, IL-1β and injected into the peritoneal cavity of C57Bl/6 mice. (D) Histological sections showing tumor cells in peritoneal lymph nodes. Data are presented as the mean ± standard error of the mean. IL, interleukin; TNF, tumor necrosis factor; SEM, standard error of the mean.

Experimental data support the hypothesis that inflammatory cytokines may either inhibit or facilitate tumor cells depending on their concentration. Fig. 2A and B show a dose-response curve demonstrating that low levels of IL-1β and TNF-α have minimal effect on cell viability, whereas when the levels of IL-1β and TNF-α increase, a significant reduction in cell proliferation and an increase in cell death is promoted in melanoma cells, either in the absence or in the presence of the pro-apoptotic signal, H_2_O_2_. In the present study, following stimulation with low doses of IL-1β and TNF-α, the melanoma cells were observed to exhibit an increased capacity to colonize the peritoneal lymph nodes, as demonstrated by the weight of the lymph nodes collected from the peritoneal cavities of the injected mice, as well as histological examination of the lymph nodes (Fig. 2C and D). These findings suggest that macrophage IL-1β and TNF-α are responsible for VEGF-C expression in TAMs, which may lead to lymphangiogenesis and melanoma lymph node metastasis.

## Discussion

Cancer cells acquire the capacity to recruit various stromal cells, including endothelial precursor cells, fibroblasts and monocytes during their progression to malignancy. Monocytes are recruited from the peripheral blood in response to tumor-derived chemokines, including monocyte chemotactic protein-1, which stimulate adhesion to endothelial cells, transendothelial migration and differentiation into macrophages (16). Macrophages in tumors are termed TAMs and are versatile cells which may reversibly express diverse functional states under the influence of host inflammatory cells and tumor cells. TAMs may function as effector cells against tumor growth, or may promote tumor aggression through releasing growth factors, cytokines and proteinases (17,18). TAMs are also the major contributor to tumor angiogenesis through the secretion of VEGF-A and have a crucial role in lymphangiogenesis through the secretion of VEGF-C.

The present study shows that, inflammatory macrophages co-cultivated with B16 murine melanoma cells express enhanced VEGF-C mRNA which was found to be associated with an autocrine loop of activity between macrophage IL-1β and TNF-α and their receptors. Furthermore, antibodies against the IL-1β and TNF-α receptors, which were added to the media conditioned by macrophages and tumor cells, were found to inhibit the enhancement of VEGF-C expression in macrophages co-cultivated with tumor cells. Thioglycollate-elicited peritoneal macrophages are inflammatory macrophages which release no, or very low quantities of TNF-α and IL-1β under unstimulated conditions. Thus, it is possible that contact with tumor cells promotes a more advanced state of activation in these macrophages. However, in the present study, this level of activation was not observed to be cytotoxic for tumor cells, as the viability of the tumor cells collected from the macrophage-tumor cell co-cultures was not significantly different compared with that of the cells grown in standard conditions. Schoppmann *et al* (9) revealed that a particular subfraction of cluster of differentiation 14-positive and VEGFR-3-expressing monocytes isolated from the peripheral blood of patients with cervical cancer, acquire the capacity to express VEGF-C following stimulation with TNF-α. By contrast, it has also been reported that macrophages co-cultivated with colon or breast carcinoma cells do not exhibit an increase in VEGF-C mRNA expression (19). It is possible that VEGF-C expression in TAMs may be associated with the different tumor histotype. It has been demonstrated that TNF-α and IL-1β may stimulate VEGF-C expression in human fibroblasts and vascular endothelial cells primarily through nuclear factor κ-light-chain-enhancer of activated B cells (20). In addition, an unexpected inducer of VEGF-C expression in human melanoma cells was the low extracellular pH (21), which has been demonstrated to enhance IL-1β production by monocytes (22).

In the present study, TNF-α and IL-1β were found to cooperate to promote the colonization of peritoneal lymph nodes by murine melanoma cells, when they were used at a non-toxic dose. A high dose of TNF-α and IL-1β was found to induce tumor cell cytotoxicity and reduce tumor cell resistance to the apoptotic agent H_2_O_2_, in a synergistic manner. TNF-α and IL-1β are the most potent pro-inflammatory cytokines produced by TAMs which, depending on their concentration, may either induce the expression of several genes resulting in the promotion of tumor cell aggression, or exert a cytotoxic effect. In tumor cells, IL-1β induces the secretion of growth- and invasion-promoting factors, matrix-metalloproteases and angiogenic molecules, including VEGF-A and basic fibroblast growth factor (23). Vidal-Vanaclocha *et al* (24) showed that IL-1β promotes experimental liver metastasis in B16 melanoma cells, while a reduction in liver metastases was observed following treatment with IL-1Ra. Moreover, experimental models of invasion and metastasis have shown that TNF-α promotes melanoma dissemination (25). However, hemorrhagic necrosis of tumors following TNF-α treatment was common and high doses of TNF-α had to be injected locally and repeatedly (25). While IL-1β is not generally considered to be a cytokine which mediates cytotoxic activity, a previous study found that IL-1β had a direct cytotoxic effect on tumor cells, with human monocyte-derived IL-1β found to be growth-inhibitory and cytocidal in A375 melanoma cells (26).

In conclusion, the present study has shown that macrophage IL-1β and TNF-α may promote VEGF-C expression in TAMs and may have a role in melanoma lymph node colonization. Targeting the signaling between TAMs and tumor cells in an inflammatory environment may be critical for inhibiting the progression of melanoma cells.

## Figures and Tables

**Table I:** Primer sequences used for reverse transcription polymerase chain reaction analysis.

Gene	Abbreviation	Forward primer 5′-3′	Reverse primer 5′-3′	Tm (°C)
Transforming growth factor beta	TGF-β	GGCTTCTAGTGCTGACG	GGGTGCTGTTGTACAAAG	54
Tumor necrosis factor alpha	TNF-α	GCGGTGCCTATGTCTCAGCC	TGAGGAGCACGTAGTCGGGG	60
Vascular endothelial growth factor C	VEGF-C	CCATGCACTTGCTGTGCTTC	ACCGGCAGGAAGTGTGATTG	59
Interleukin 1 beta	IL-1β	CCTGCAGCTGGAGAGTGTGGA	CCCATCAGAGGCAAGGAGGAA	60
Beta 2 microglobulin	β2m	TGCTATCCAGAAAACCCCTC	GTCATGCTTAACTCTGCAGG	58
Interleukin 1 receptor 1	IL-1R1	ACCCCCATATCAGCGGACCG	TTGCTTCCCCCGGAACGTAT	58
Tumor necrosis factor alpha receptor 1	TNFaR1	GGATACAGTCTGCAGGGAGTG	TCCACCGGGGATATCGGCACATTAA	60
Transforming growth factor beta receptor 1A	TGFβR1A	GAGCTCTGCAGTTGAGACGTTTAG	AACAAAACACTGCTTTGATCAAGTA	58
